# 胸腔注射榄香烯致严重不良反应7例病例系列分析

**DOI:** 10.3779/j.issn.1009-3419.2018.06.06

**Published:** 2018-06-20

**Authors:** 菲 高, 宜 邵, 殿胜 钟, 夏 刘, 凡路 孟

**Affiliations:** 300052 天津，天津医科大学总医院肿瘤科 Department of Medical Oncology, Tianjin Medical University General Hospital, Tianjin 300052, China

**Keywords:** 榄香烯注射液, 胸腔注射, 不良反应, 恶性胸腔积液, 呼吸困难, Elemene injection, Chest injection, Adverse reactions, Malignant pleural effusion, Dyspnea

## Abstract

**背景与目的:**

恶性胸腔积液（malignant pleural effusion, MPE）指胸膜原发恶性肿瘤或其他部位恶性肿瘤转移至胸膜引起的胸腔积液。胸腔内注射榄香烯对MPE有良好的治疗效果，在临床中应用较普遍，同时存在一些不良反应，但文献缺乏对严重不良反应的报道及处理。本研究旨在观察榄香烯注射液治疗恶性胸腔积液导致不良反应发生情况，探讨严重不良反应发生原因和应对方案。

**方法:**

回顾性分析14例胸腔注射榄香烯病例，对其中7例发生严重不良反应病例进行详细报道。

**结果:**

榄香烯注射液胸腔注射所致严重不良反应主要表现为剧烈胸痛、呼吸困难、喘憋、意识模糊和凝血功能障碍，经临床对症治疗后均可缓解。

**结论:**

胸腔注射榄香烯榄香烯对MPE有一定治疗价值，使用时应严格把握适应证、筛选患者、严密监测。

## Abstract (en)

**Background and objective:**

Malignant pleural effusion (MPE) refers to pleural effusion which arises from primary malignant tumor of pleura or other pleural metastatic tumors. Injection of elemene in chest makes good effect on the treatment of MPE, and is widely used in clinic. Adverse effects also exist, but the severe adverse effects and relevant managements are rarely reported. The aim of this study is to observe the adverse reactions induced by the treatment of malignant pleural effusion through elemene injection and to explore the solutions.

**Methods:**

A retrospective analysis was made on 14 cases of patients receiving intra-pleural injections with elemene, and the incidence of severe adverse reactions of 7 cases were disscussed in detail.

**Results:**

Most of the severe adverse reactions caused by elemene were severe chest pain, dyspnea, wheezing, clouding of consciousness and coagulopathy.

**Conclusion:**

Strict screening, full preprocessing and close monitoring are necessary to prevent serious adverse reactions caused by elemene injection in the treatment of malignant pleural effusion.

恶性胸腔积液（malignant pleural effusion, MPE）指胸膜原发恶性肿瘤或其他部位恶性肿瘤转移至胸膜引起的胸腔积液。几乎所有的恶性肿瘤均可导致MPE的产生，肺癌最常见，约占MPE的1/3，其次是乳腺癌。5%-10%的MPE找不到原发病灶^[1]^。MPE具有增长速度快、复发率高、血性居多等临床特点，一旦出现通常提示原发部位病变已经局部转移或全身扩散，预后较差，中位生存期仅3个月-12个月。目前主要采用全身化疗结合局部治疗，即在针对原发肿瘤病灶化疗同时，加用胸膜固定术，在胸水引流基础上向胸膜腔内注入硬化剂造成胸膜粘连^[2]^。但对硬化剂的选择尚无强有力的循证医学支持，安全性和有效性缺乏大样本随机对照研究^[3]^。此外，如何治疗和控制恶性胸腔积液发展及复发，仍是临床工作中的难题。

榄香烯（elemene）是我国自行开发的从姜科植物温郁金中提取的有效抗癌成分。相关药理学研究证实，榄香烯除直接杀伤肿瘤细胞外，其在胸腔内注射后可使脏壁胸膜间发生化学炎症，诱导胸膜粘连、胸膜腔闭塞，从而控制MPE发生^[4]^。目前临床报道榄香烯乳使用过程中不良反应以静脉炎为主，为静脉滴注中发生，而胸腔注射不良反应多为疼痛和发热，可自然缓解或经简单对症治疗后好转^[5, 6]^，缺乏严重不良反应的深入研究及应对方案。本研究旨在回顾性分析胸腔注射榄香烯治疗MPE过程中出现的7例较严重不良反应，提出相关改良措施，具体报道如下。

## 1 资料与方法

### 1.1 临床资料

选择天津医科大学总医院肿瘤科2016年6月-2017年8月，经病理和/或细胞学证实的恶性肿瘤，胸水或沉渣中找到肿瘤细胞证实为恶性胸腔积液，采用胸腔注射榄香烯注射液（大连金港制药有限公司）的患者14例，其中男性6例，女性8例，平均年龄63岁。卡氏评分60分以上，预计生存期 > 3个月，无明显心肺肝肾等重要器官功能障碍。其中，发生严重不良反应7例，男性5例，女性2例，年龄49岁-77岁，平均年龄60.7岁。原发肿瘤为肺癌4例，胃癌1例，乳腺癌1例，原发性腹膜癌1例。其中3例有肺栓塞病史，1例有慢性阻塞性肺疾病病史，1例存在青霉素及磺胺类药物过敏史。

### 1.2 给药方式

患者行胸腔置管引流，2 d-4 d待胸水排净后，先予2%利多卡因10 mL注入胸腔，10 min-15 min后使用榄香烯注射液100 mg加生理盐水100 mL缓慢注入胸腔，观察患者临床相关胸痛、胸闷等不良反应发生情况。若30 min后无明显不良反应，则继续将剩余剂量榄香烯注射液加生理盐水100 mL缓慢注入胸腔。（榄香烯注射液用量按300 mg/m^2^计算）。夹闭导管，嘱患者每15 min-30 min变更体位，使药物与胸膜充分接触，闭管24 h后再次开放引流管。

### 1.3 治疗评效

效果评估（1个月-3个月）主要采用胸部计算机断层扫描（computed tomography, CT）、B超检查方式，参照世界卫生组织（World Health Organization, WHO）胸腔积液评估标准分为：完全缓解（complete response, CR）:积液完全消失、持续4周以上; 部分缓解（partial response, PR）：积液较治疗前减少50%以上，持续4周; 疾病稳定（stable disease, SD）：积液较治疗前减少不足50%，或增加不超过25%;疾病进展（progressive disease, PD）：积液较治疗前增加超过25%以上。以（CR+PR）计算有效率。

## 2 结果

### 2.1 治疗评效

14例患者中，3例患者失访，11例可评估患者中，PR：6例，SD：4例，PD：1例，有效率54.5%。

### 2.2 不良反应

#### 2.2.1 一般不良反应

14例患者中，2例出现发热，为中低热，发生于用药后2 h-4 h，经物理降温、非甾体类药物对症处理后，体温在2 h内均可降至正常; 5例出现胸痛，疼痛评分多为3分-5分，予曲马多、吗啡等止痛后，均可得到缓解。

#### 2.2.2 严重不良反应

14例患者中，7例患者出现严重不良反应，均为首次用药。1例表现为呼吸困难、紫绀、意识模糊; 2例表现为剧烈胸痛; 3例表现为呼吸困难、喘憋; 1例表现为凝血功能障碍。发生时间为用药后20 min-14 h，见表 1。

**1:** 7例患者临床资料及不良反应发生情况 Clinical data and adverse reactions of 7 patients

Case number	Sex	Age (yr)	Primary tumor	Symptoms	Abnormal signs	Appearance	Previous history
#1	F	49	Gastric adeno- carcinoma	Dyspnea, clouding of consciousness	Cyanosis; BP: 85/60 mmHg; ABG: pO_2_ 65 mmHg	20 min	
#2	F	63	Lung adeno- carcinoma	Severe chest pain; NRS: 9, lasts 72 h		30 min	
#3	F	67	primary peritoneal carcinoma	Severe chest pain; NRS : 8, lasts 30 h		1 h	
#4	F	63	Breast carcinoma	Dyspnea, wheezing	Dry sounds in lungs; ABG: pO_2_ 60 mmHg	20 min	PE
#5	M	71	Lung adeno- carcinoma	Dyspnea, wheezing	ABG: pO_2_ 61 mmHg	2 h	COPD
#6	M	59	Squamous lung carcinoma	Dyspnea, wheezing		2 h	PE
#7	M	53	Lung adeno- carcinoma		PT: 94.2 s; PT-INR: 8.24	14 h	PE
NRS: numeric rating scale. The patient was evaluated on a 10-point scale based on pain. Level 0 is painless, 1-3 is mild pain, 4-6 is moderate pain, 7-10 is severe pain; F: female; M: male; ABG: arterial blood gas; PE: pulmonary embolism; COPD: chronic obstructive pulmonary disease; #3: The patient was allergic to penicillin and sulfonamides; #7: The patient was in anticoagulant therapy with warfarin, and the PT-INR was stable at 2-3 before the intra-pleural injection of elemene.							

#### 2.2.3 严重不良反应患者的处理及转归

#1患者于榄香烯注入20 min后出现严重胸闷、呼吸困难、意识模糊; 查体：皮肤紫绀，四肢湿冷，血压85 mmHg/60 mmHg，心率117 bpm; 血气提示pO_2_ 65 mmHg。考虑过敏反应，亦不除外胸膜反应，迅速给予吸氧，静脉使用地塞米松5 mg、甲泼尼龙40 mg，肌注苯海拉明及异丙嗪，症状缓解不明显，遂打开引流管引流出注射的榄香烯，症状于2 h缓解。

#2患者于榄香烯注入30 min后出现胸部剧烈疼痛，数字分级法（numeric rating scale, NRS）评分9分，予吗啡5 mg皮下注射，30 min后疼痛评分降至6分，1 h后症状复发，再次予吗啡注射液5 mg皮下注射，疼痛部分缓解。之后，予羟考酮控释片10 mg Q12h止痛。

#3患者于榄香烯注入1 h后出现胸部剧烈疼痛，NRS评分8分，予盐酸布桂嗪注射液100 mg皮下注射，1 h后疼痛评分6分，再次予曲马多100 mg肌注，疼痛部分得到控制。之后，予羟考酮控释片10 mg Q12h止痛。

基于以上3例患者出现的严重副作用，我们分析，可能与预处理不够，注入胸腔药物的速度过快有关，于是，更改了给药方案：先予2%利多卡因10 mL+NS 50 mL胸腔注射; 提前0.5 h-1 h预服羟考酮10 mg; 注药时，先予榄香烯100 mg+NS 100 mL缓慢注入，观察30 min，若无明显胸闷、胸痛不适，再将剩余榄香烯+NS 100 mL，使用输液泵调控注药速度，在监测患者反应的基础上，缓慢泵入。

#4患者于用药20 min后出现喘憋，血气分析：pO_2_ 60 mmHg，心电图、心肌酶正常，予吸氧、甲泼尼龙40 mg静脉点滴，结合既往肺栓塞病史，强化克赛抗凝治疗; 3 d后好转。

#5患者于注药2 h后出现呼吸困难、喘憋，血气分析：pO_2_ 61 mmHg，心电图、心肌酶、BNP均正常，结合既往慢性阻塞性肺病病史，考虑诱发慢性阻塞性肺病急性发作，予吸氧、甲泼尼龙40 mg静脉点滴，氨溴索、多索茶碱等治疗，症状于3 h缓解。

#6患者入院时即有喘憋不能平卧症状，注药后约2 h患者出现呼吸困难，喘憋加重，予高流量吸氧，甲泼尼龙40 mg静脉点滴，约2 h后逐渐好转。

#7患者在给药过程中即出现胸闷，给予甲泼尼龙静点缓解，完成注药。14 h后喘憋、呼吸困难较前加重，提前打开引流管，发现血性积液颜色较前加深，结合患者既往肺栓塞和长期口服华法林病史，急查凝血酶原时间（prothrombin time, PT）：94.2 s; 凝血酶原时间国际标准化比值（prothrombin time international ratio, PT-INR）：8.24，予吸氧，间断给予维生素K1 2.5 mg肌注解救，下调华法林用量，次日，PT-INR降为3.36，2 d内症状渐趋稳定。

## 3 讨论

榄香烯是从中药莪术中提取的抗肿瘤药，以β-榄香烯为主要活性成分，占60%-72%，同时含有少量α及γ-榄香烯及其它萜烯类化合物，经由对癌细胞亲和性强的乳化剂特殊处理而制成乳剂注射液^[7]^。榄香烯不但可直接抑制肿瘤细胞生长，还可影响将肿瘤细胞阻滞在G_2_/M期，减少其有丝分裂，导致受影响的肿瘤细胞快速凋亡^[8]^。榄香烯能增强肿瘤免疫原性及机体T淋巴细胞亚群的功能，发挥辅助抗肿瘤作用^[4]^。同时因为刺激性较强，注入胸腔后，刺激脏壁胸膜发生无菌性炎症反应而减少胸腔积液的形成。

既往Ⅲ期临床试验及*meta*分析表明，榄香烯注射液对恶性胸腔积液具有良好的治疗效果，有效率为56%-86.7%^[9-11]^。文献中提到胸腔注射的不良反应主要为胸痛和发热，给予非甾体抗炎药等对症治疗后，症状能够缓解，绝大多数患者均能很好的耐受。胸腔注射所致严重不良反应研究较少，仅有一些个案报道^[12-16]^，且症状描述及处理方式相对简单。本研究共报道7例胸腔注射所致严重不良反应，原因分析如下：（1）药物因素：①榄香烯注射液除了β-榄香烯有效成分外，还含有少量的γ、δ-榄香烯及其他萜烯类化合物，不良反应可能和原料本身性质、杂质含量等有关^[17]^;由于榄香烯可刺激血管扩张，也可引发过敏反应或血管迷走性晕厥^[6]^;②莪术属活血化瘀类中药，榄香烯注射液说明书注明对血小板减少症或有进行性出血倾向者慎用，动物试验表明，β-榄香烯可延长血瘀大鼠凝血酶原时间（prothrombin time, PT）及凝血酶时间（thrombin time, TT），通过抑制血小板聚集和（thromboxane A2, TXA2）的释放发挥抗血栓作用^[18, 19]^;同时，临床静脉应用榄香烯发现，其具有一定的抑制血小板聚集、粘附，降低纤维蛋白浓度及全血粘度的作用^[6]^;而华法林为双香豆素类抗凝剂，通过竞争性对抗维生素K，抑制肝细胞凝血因子的合成，还可降低凝血酶诱导的血小板聚集反应。胸腔注射榄香烯，可部分吸收入血，推测榄香烯与华法林存在协同作用，同时，榄香烯可能延长华法林体内代谢时间，进而进一步增强其抗凝效果，甚至导致出血。（2）预处理不足及注药速度过快：榄香烯注射液对胸膜具有较强的刺激作用，高浓度的药物会直接、持续性地刺激壁层胸膜上的神经末梢，引起疼痛，尤其在首次给药后容易发生。（3）患者因素：发生严重不良反应的患者多为晚期肿瘤病人，一般状况不佳，多伴有合并症及过敏史。且榄香烯属挥发油类，脂溶性强，肺组织中浓度最高，从呼吸道排出及体内生物转化是其主要消除途径^[7]^。合并慢性阻塞性肺病、哮喘等气道疾病患者可能对其耐受性差。

榄香烯胸腔注射亦可导致严重不良反应发生，因此临床医师应提高对其不良反应的认识。整理我科胸腔注射榄香烯发生严重不良反应病例，总结几点经验如下：（1）临床使用时应严格掌握适应证和用法用量。慎用于凝血功能严重异常、血小板减少、有进行性出血倾向的患者。（2）对于一般状况差，合并肺部疾患和具有过敏史患者需格外小心，文献中尚有榄香烯胸腔注入致肺水肿、呼吸衰竭的个案报道^[12, 13]^，因此用药期间应密切观察患者症状体征变化，发生不良反应时迅速处理。（3）预处理方面，建议先予利多卡因加生理盐水充分局麻胸腔，对于体弱、女性患者必要时予阿片类控释药物预服防止剧烈胸痛发生。胸腔内注入地塞米松预处理虽然有可能降低不良反应，但可能影响化学性胸膜炎产生，降低胸水控制效果，故不建议使用^[20]^。（4）给药时，可使用输液泵控制液体速度，缓慢泵入。

总体来讲，榄香烯注射液是一个安全、有效、经济的辅助治疗药品，只要重视预防措施，密切关注患者用药后反应，积极地应对出现的不良反应，在恶性胸腔积液治疗方面还是有着良好的应用前景。
